# HAT: *de novo* variant calling for highly accurate short-read and long-read sequencing data

**DOI:** 10.1093/bioinformatics/btad775

**Published:** 2024-01-04

**Authors:** Jeffrey K Ng, Tychele N Turner

**Affiliations:** Department of Genetics, Washington University School of Medicine, St Louis, MO 63110, USA; Department of Genetics, Washington University School of Medicine, St Louis, MO 63110, USA

## Abstract

**Motivation:**

*de novo* variants (DNVs) are variants that are present in offspring but not in their parents. DNVs are both important for examining mutation rates as well as in the identification of disease-related variation. While efforts have been made to call DNVs, calling of DNVs is still challenging from parent–child sequenced trio data. We developed **H**are **A**nd **T**ortoise (HAT) as an automated DNV detection workflow for highly accurate short-read and long-read sequencing data. Reliable detection of DNVs is important for human genomics and HAT addresses this need.

**Results:**

HAT is a computational workflow that begins with aligned read data (i.e. CRAM or BAM) from a parent–child sequenced trio and outputs DNVs. HAT detects high-quality DNVs from Illumina short-read whole-exome sequencing, Illumina short-read whole-genome sequencing, and highly accurate PacBio HiFi long-read whole-genome sequencing data. The quality of these DNVs is high based on a series of quality metrics including number of DNVs per individual, percent of DNVs at CpG sites, and percent of DNVs phased to the paternal chromosome of origin.

**Availability and implementation:**

https://github.com/TNTurnerLab/HAT

## 1 Introduction

*de novo* variants (DNVs) are variants present in offspring but not in their parents (Kong *et al.* 2012). These “new” variants are present in every individual and on average each individual has ∼40–100 DNVs within their genome. Common characteristics of DNVs include ∼20% occurring at CpG sites and ∼75% originate on the paternal chromosome of origin (Ng *et al.* 2022). To date, DNV calling methods have primarily focused on whole-exome sequencing (WES) and whole-genome sequencing (WGS) from short-read sequencers (Iossifov *et al.* 2014, Turner *et al.* 2017). This is because the majority of parent–child sequenced trios are from short-read sequencing. In short-read sequencing WES and WGS, reliable detection of DNVs can be obtained from regions of the genome with good mappability (Turner *et al.* 2017). We first implemented and optimized Hare-And-Tortoise (HAT) on Illumina short-read WGS data (Ng *et al.* 2022). However, with the rapidly expanding WES datasets (Feliciano *et al*. 2018), we focus our attention on adding a feature to HAT for optimizing DNV calling in Illumina short-read WES data. Furthermore, in 2019, highly accurate long-read sequencing data (i.e. PacBio HiFi) became available and is enabling novel insights into more challenging regions of the genome (Wenger *et al.* 2019). We further optimize HAT to work on this data type as well.

In this article, we introduce HAT as a DNV caller optimized for sequencing data from Illumina short-read WES, Illumina short-read WGS, and PacBio HiFi long-read WGS in parent–child sequenced trios. HAT is important for generating DNV calls for use in studies of mutation rates (Ségurel *et al.* 2014) and identification of disease-relevant DNVs (Iossifov *et al.* 2014). Unlike most DNV callers, the ability to call DNVs from multiple sequencing types is significant. We are aware of only one other DNV caller that can work on these three data types and that is DeepTrio implemented in Google’s DeepVariant (Kolesnikov *et al.* 2021). Our comparisons in this study show the utility of both DeepTrio and HAT for detection of DNVs.

We rely on four main data resources in this paper. The first dataset is a set of 100 parent–child sequenced trios with Illumina short-read WES from the SPARK cohort (Feliciano *et al*. 2018), the second dataset is a set of 4216 trios with Illumina short-read WGS from the Simons Simplex Collection (Ng *et al.* 2022), the third dataset is PacBio HiFi long-read WGS from trios with neurodevelopmental disorders (Mehinovic *et al.* 2022, Sams *et al.* 2022), and the fourth dataset is Illumina short-read WGS and PacBio HiFi long-read WGS from the gold standard Genome In A Bottle (GIAB) trio (Krusche *et al.* 2019). In particular, the GIAB trio is a benchmark dataset by many researchers worldwide for assessing variation. By application of HAT to each of these datasets, we show that high-quality DNVs are attainable with all three sequencing types.

## 2 Materials and methods

### 2.1 The HAT workflow

**Figure 1.**
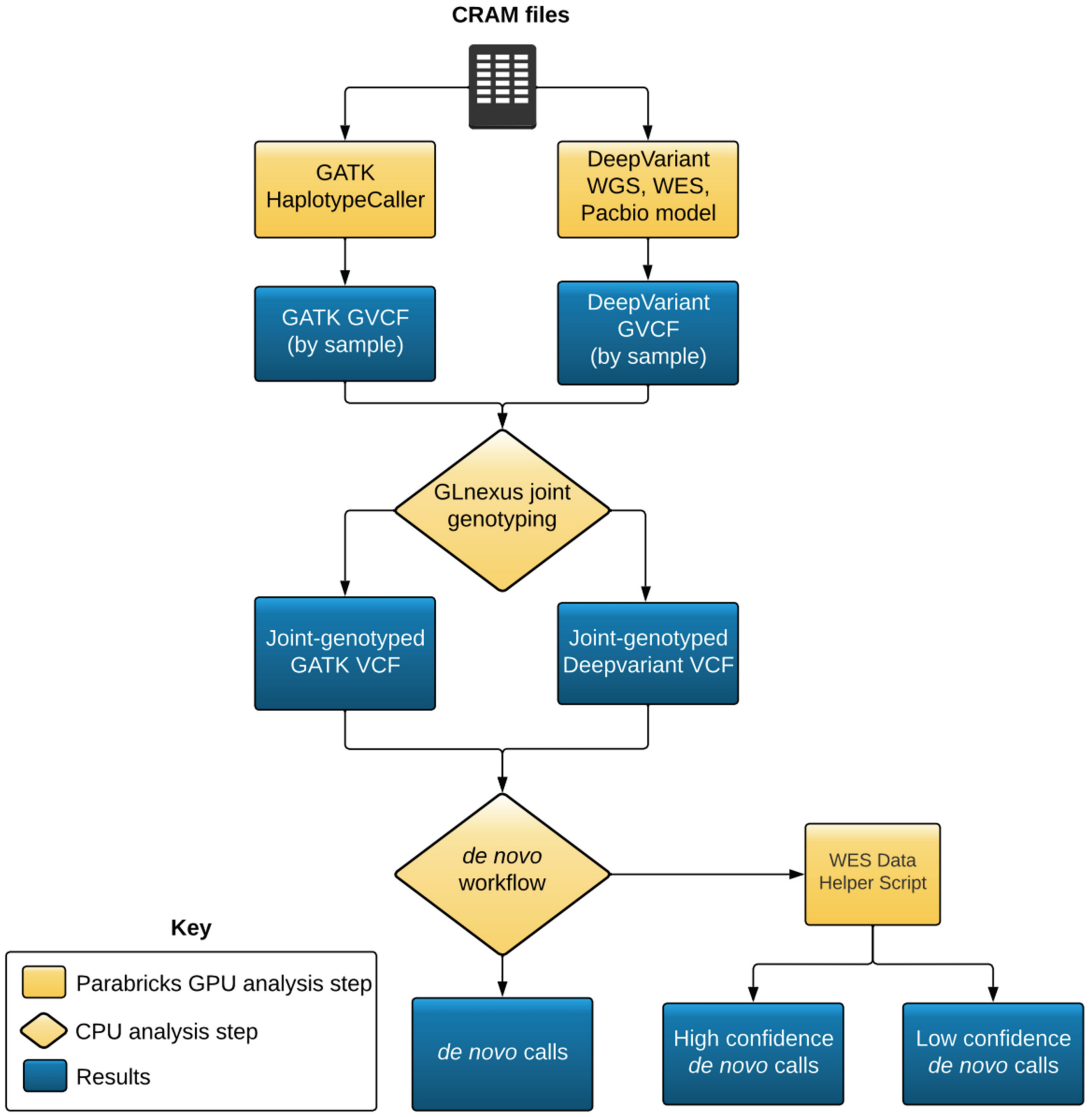
HAT workflow schematic. This figure shows how the general workflow of how HAT works

The HAT workflow consists of three main steps: GVCF generation, family-level genotyping, and filtering of variants to get final DNVs (Fig. 1).

We leveraged the freely available, GPU accelerated NVIDIA Parabricks (Franke and Crowgey 2020) software (v4.0.0-1) for rapid GVCF generation, specifically GATK HaplotypeCaller (McKenna *et al.* 2010, Poplin *et al.* 2018) and DeepVariant (Poplin *et al.* 2018). We also provide a purely CPU-based option, Tortoise, which uses open-source versions of GATK and DeepVariant. The genotyping step is performed with GLnexus (Yun *et al.* 2020) and post-genotyping filtering is done with our custom workflow. The default filtering steps include the following: (i) a requirement that the variant is seen in the child with a genotype of 0/1 or 1/1, (ii) a requirement that the parents have a genotype of 0/0, (iii) the variant must be in the intersection of GATK and DeepVariant, (iv) the depth of coverage at the site must be at least 10×, (v) the quality of the genotype of the variant in the child must be at least 20, (vi) the variant allele must be found in at least 25% of the reads, (vii) the parents cannot contain any reads with the variant, and (viii) the variants in recent repeats, low complexity regions, and centromeres are filtered out. When running HAT on WES data, we offer a follow-up workflow to separate DNV calls into high and low confidence regions. Assuming the capture region has a buffer of 50 bp on each end, we consider DNVs found within the capture region ±10 bp to be high confidence calls and the DNVs at the end of the capture region low confidence. After defining the high and low confidence areas of each capture region, the code will look at Samtools mpileup output and count how many times the alternate allele appears in the parents and the child. By default, if the alternate does not appear at all, at any quality level, in the parents and at least once in the child the DNV will be in the final high confidence callset.

HAT is capable of running on Docker (Merkel 2014) compatible machines and high-performance clusters, as well as in the cloud. We offer the workflow as both a Snakemake (Koster and Rahmann 2012) and a Cromwell workflow https://cromwell.readthedocs.io/en/stable/, respectively. The total run time for Hare with WGS data, assuming four V100 or A100 GPUs, is 4.5 h. For WES data, only one GPU V100 or A100 is needed with a total parallelized run time of seven minutes. Lastly, Tortoise has been optimized to run on PacBio data by switching the model type for DeepVariant to “PACBIO” and has an overall runtime of 2.5 days because it does not use GPU acceleration. In the tested version of Parabricks, GATK is not supported for PacBio.

### 2.2 Sample collections and DNV calling

Illumina short-read WES samples (IDT xGen exome capture) included 100 trios from the SPARK Collection. One set of the Illumina short-read WGS samples included 4216 trios from the Simons Simplex Collection. The alignment data for both of these collections was accessed through SFARI Base and downloaded to our LSF server for running HAT. The other set of Illumina short-read WGS was the Genome In A Bottle trio (HG002-HG003-HG004) available from https://github.com/genome-in-a-bottle/giab_data_indexes/blob/master/AshkenazimTrio/alignment.index.AJtrio_Illumina300X_wgs_novoalign_GRCh37_GRCh38_NHGRI_07282015. PacBio HiFi long-read samples included one trio from our previous publication on 9p Minus Syndrome (Sams *et al.* 2022), three trios from our previous publication on autism (Mehinovic *et al.* 2022), and the same Genome In A Bottle trio as mentioned above. HAT was run on our server using the Snakemake workflow using both V100 and A100 GPUs for acceleration. When running DeepVariant on the HG002 PacBio trio, we used the PacBio Revio model provided by PacBio found here: https://downloads.pacbcloud.com/public/revio/2022Q4/dv-model/.

### 2.3 Downsampling of 300× Illumina data and 9p PacBio long-read data

After downloading the 300× Illumina WGS HG002 trio data, we realigned the trio to reference file GCA_000001405.15_GRCh38_no_alt_analysis_set.fasta with SpeedSeq (Chiang *et al.* 2015) and then downsampled the 300× samples using the “samtools view –subsample” command, with a subsample seed of 69 (Li *et al.* 2009) to around ∼30× coverage. The subsampled trio data was then run through HAT as previously described. For the 9p PacBio long-read trio data, we used the same Samtools command and random seed, first subsampling down to a coverage level of 10×, 20×, and 30×. We subsequently subsampled the 9p trio 100 times each, down to a coverage level of 20× and 30×. The subsample seeds used were 0–99 for the 100 different replications, respectively, for both 20× and 30×.

### 2.4 *de novo* variant confirmation using 300× Illumina WGS HG002 data

We used the Samtools “mpileup” command over all of the DNVs detected on the 300× Illumina WGS data of the HG002 trio from Genome In A Bottle. We counted the occurrences of each alternate allele. If the DNV had a variant allele frequency of >25% in the child and <1% variant allele frequency in the parents, the DNV is considered confirmed based on the high coverage data. We used a 25% variant allele frequency threshold in the child because it is the default allele frequency cutoff used in HAT. We used a 1% variant allele frequency cutoff in the parents because it corresponds to the error rate found in Illumina sequencing (Stoler and Nekrutenko 2021).

## 3 Results

### 3.1 DNVs from short-read WES

We tested HAT on 100 trios from the SPARK collection (Feliciano *et al*. 2018). After initial detection of DNVs, a specific filtering script trims provided WES capture regions to sort DNVs into high and low confidence calls and marked as such in the output file (Supplementary Fig. S1). We found 282 DNVs, of which 190 were found in high confidence regions and 92 were low confidence (Supplementary Table S1). In the high confidence callset, the DNV confirmation rate was 91.6% as compared to 70.7% in the low confidence callset with DNV features within expectations (Table 1).

**Table 1.** *de novo* variants detected in WES and WGS datasets.^a^

Data type	*de novo* variants	Percent of *de novo* variants in CpG regions	Ti/Tv ratio
WES	2 ± 1	34.7%	2.39
WGS	78 ± 15	18%	2.11

a This table shows *de novo* variants metrics from HAT on a 100 trio WES SPARK dataset and a 4216 WGS trio dataset from the Simons Simplex Collection.

### 3.2 DNVs from short-read WGS

We previously tested HAT on 4216 trios, with DNA derived from blood, from the Simons Simplex Collection (Ng *et al.* 2022). Overall, we identified a total of 329 589 DNVs and observed that all the DNV values fall in line with expectations (Table 1), illustrating that this pipeline detects high-quality DNVs from short-read WGS. For comparison to a known control data, we also ran HAT on the 300× HG002 trio from Genome In A Bottle. We first downsampled the data to ∼30× and then ran HAT. We discovered 1105 DNVs from this trio (Supplementary Table S2). The data had ∼16.5% of the DNVs found within a CpG region. The spike in DNVs can likely be explained by cell line artifacts, as seen previously (Ng *et al.* 2022).

### 3.3 DNVs from long-read WGS

**Figure 2.**
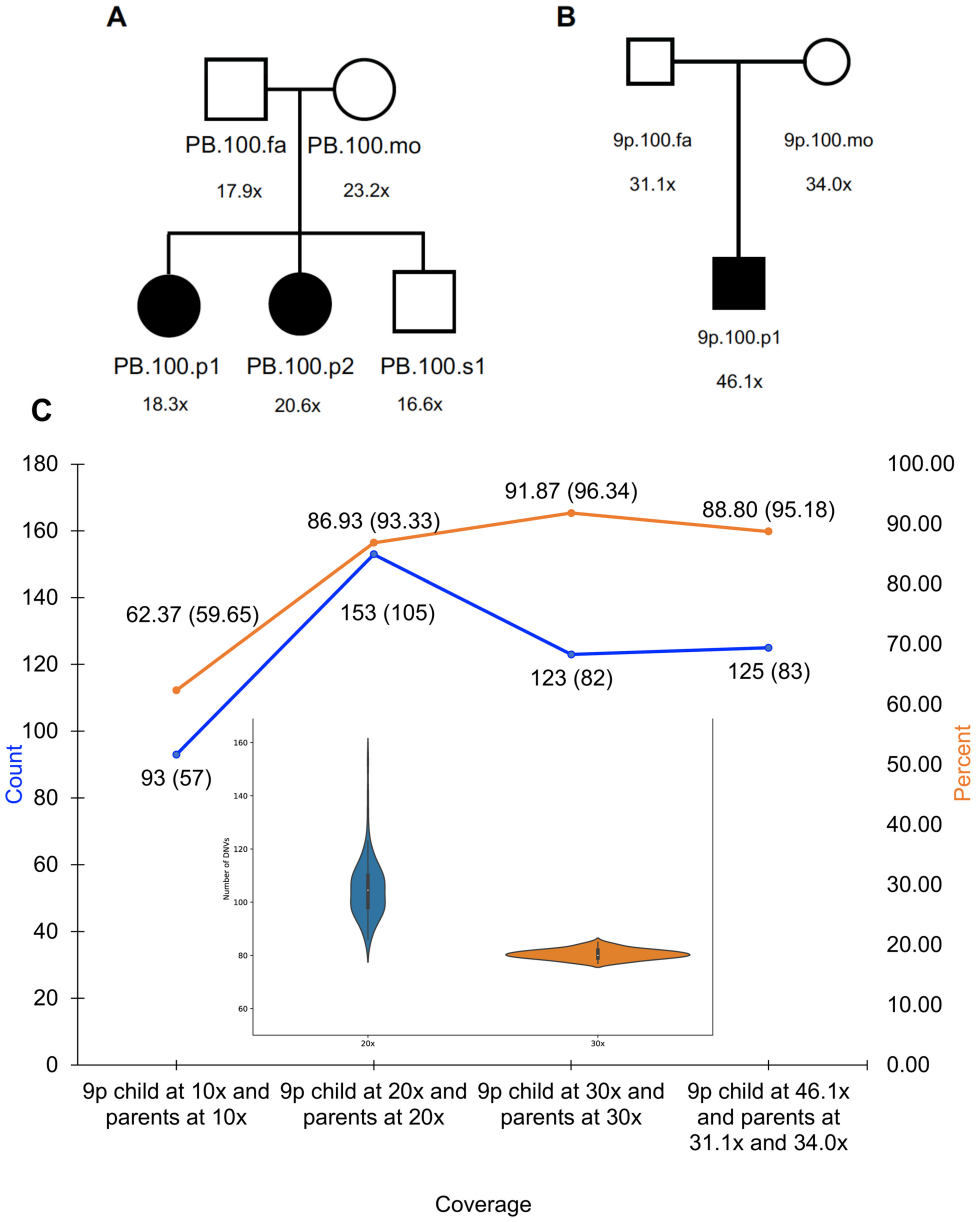
Long-read sequencing families and DNVs called with HAT. (A) Pedigree of the PB.100 family, with long-read sequencing coverage shown. This family was sequenced by PacBio HiFi sequencing in Mehinovic *et al.* (2022). (B) Pedigree of the 9p.100 family, with long-read sequencing coverage shown. This family was sequenced by PacBio HiFi sequencing in Sams *et al.* (2022). (C) This graph illustrates the increase in the quality of DNV calls as the coverage increases in the downsampled 9p.100 family. The percent of DNVs confirmed, shown in orange, increases with coverage. The percentage in parenthesis is the percent of confirmed DNVs in unique regions of the genome. The total DNV count, shown in blue, is around expected as coverage increases. The counts in parenthesis are the number of DNVs in unique regions of the genome. The violin plot shows the distribution of DNVs at 20× and 30× from 100 downsamplings of the trio WGS data

We ran HAT on four different PacBio HiFi long-read sequenced trios (Mehinovic *et al.* 2022, Sams *et al.* 2022) (Fig. 2A and B, Supplementary Table S3).

Long-read sequencing allows for more accurate DNV detection in repeat regions (Noyes *et al.* 2022). HAT found ∼94 DNVs per trio in unique regions, ∼62 per trio in repeat regions, with a total of ∼156 DNVs found per trio (Supplementary Table S2). After manual inspection of the DNVs, the confirmation rate was lower than our initial expectations (Supplementary Table S4). When assessing fold coverage of the genome in the families, the 9p.100 family had the highest confirmation rate as well as being the most deeply sequenced (Fig. 2A and B). This family had DNV metrics within our expectations (Table 2).

**Table 2.** *de novo* variant metrics of 9p.100.p1.^a^

Family	*de novo* variants	Percent of *de novo* variants in CpG regions	Ti/Tv ratio
9p.100.p1	125	21.1%	2.11

a This table shows the *de novo* variant metrics for the long-read sequenced family 9p.100.p1.

From this analysis, we hypothesized our lower-than-expected DNV confirmation rate was due to lower coverage seen in the PB.100 family.

To test this hypothesis, we downsampled the 9p.100 family to ∼10×, ∼20×, and ∼30× for each individual and reran HAT. As the coverage increased, the confirmation rate increased from 62.4% to 91.9% in 30× coverage (Fig. 2C) with a confirmation rate of 96.3% in variants residing in unique regions of the genome. To further our assessment of the quality of DNVs at 20× and at 30×, we performed downsampling 100 times at each of these depths. We found 105 ± 11 DNVs in the 20× downsamplings and 80 ± 2 in the 30× downsamplings (Fig. 2C). From these analyses, we conclude that 30× coverage genomes, in each of the members of the parent–child sequenced trio, are required for accurate DNV calling from highly accurate long-read WGS data.

As we did for the short-read WGS, we also ran HAT on the HG002 trio from Genome In A Bottle, using two replicates of PacBio HiFi data. HAT called 1108 DNVs from one replicate of the HG002 trio and 1106 DNVs from the second replicate (Supplementary Table S2). The percent of DNVs found at a CpG site was ∼16.6% for the first replicate and ∼17.0% for the second replicate.

### 3.4 Comparison of DNVs detected by HAT to DNVs detected by DeepTrio

**Figure 3.**
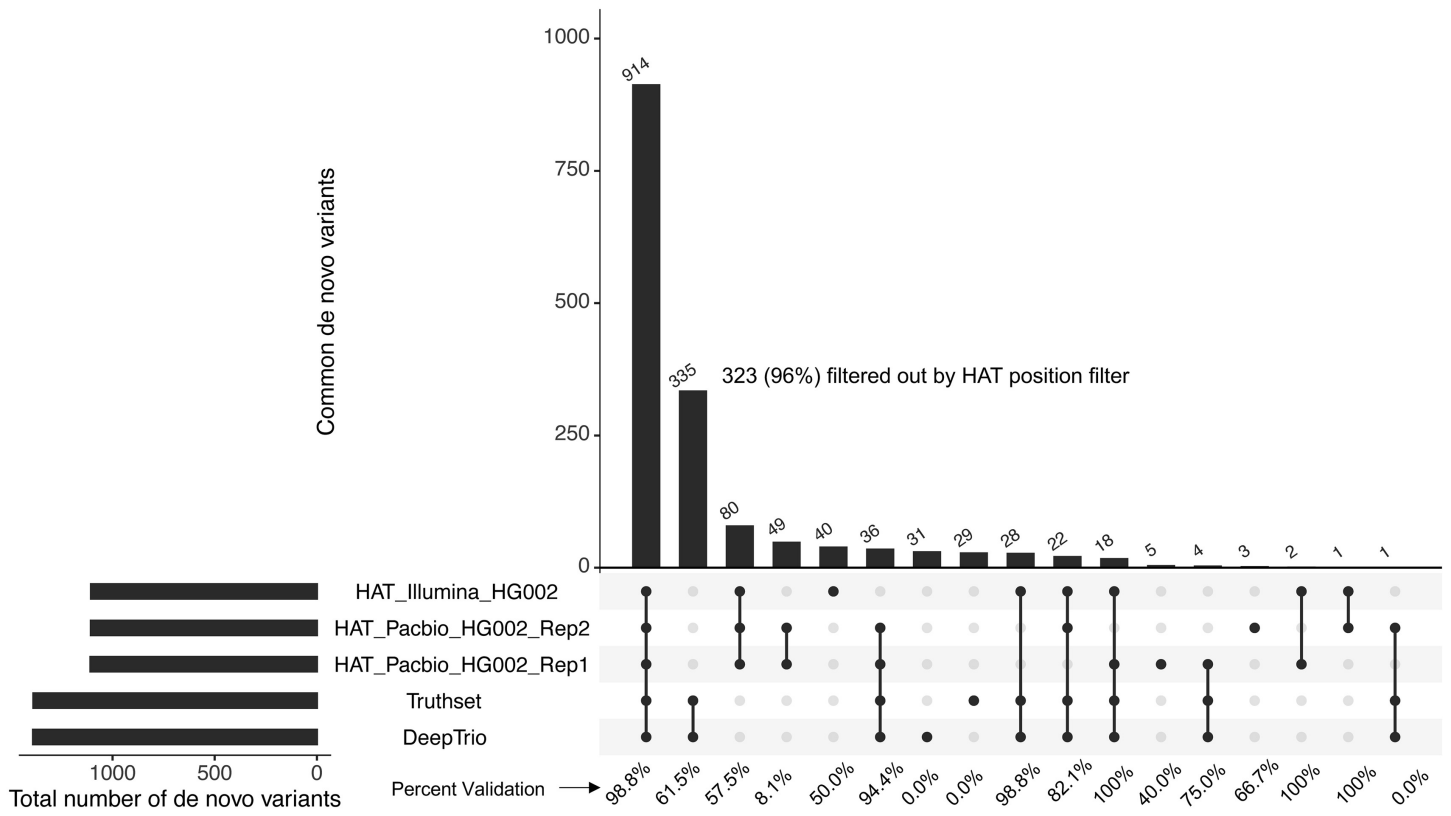
Upset plot of HG002 DNV data. This is an upset plot illustrating the overlap of DNVs between five different datasets of the HG002 trio. The datasets include HAT run on HG002 Illumina WGS and two replicates of PacBio HiFi long reads. The truthset and the DeepTrio datasets were run on Illumina WGS. The numbers above the histogram on the main figure show the total number of DNVs. The percentage underneath the various groups show the percent confirmation of DNVs by the 300× data

To test how HAT compares to other DNV callers, we specifically examined DNV calls made using the tool DeepTrio on the Illumina WGS data (obtained via personal communication with Dr Andrew Carroll) on the Genome In A Bottle Trio HG002. We also compared these to the Genome In A Bottle truth datasets (also obtained via personal communication with Dr Andrew Carroll). We considered 30× Illumina short reads, two replicates of HG002 PacBio HiFi long reads, and the truthset when comparing HAT DNV calls to DeepTrio. When compared all of the data, we found 914 DNVs overlapped (Fig. 3) between all five DNV callsets, with 96% of these confirmed by the 300× data.

There were 335 DNVs found by DeepTrio and the truthset that HAT did not call. Of the 335, only 12 DNVs were found in regions that HAT does not blacklist during the default filtering process. Looking at the mpileup count data from the full 300× WGS HG002 trio, only 2 of the 12 were confirmed from the 300× mpileup results (variant allele frequency <1% in the parents, >25% in the child). Based on this result, we hypothesized that there could be many DNVs that DeepTrio calls but HAT does not due to our position blacklist. We then ran the same position filters on the DeepTrio callset and saw a ∼94% (982 DNVs) overlap to our Illumina WGS callset. Of these 982 DNVs, 98% of the DNVs were confirmed by the 300× data. DeepTrio found 56 unique DNVs, 73% were confirmed. HAT found 123 unique variants, 56% were confirmed by the 300× data. We also saw very high overlap of ∼93% between the two PacBio HiFi replicates and the filtered DeepTrio results. Overall, after filtering regions of the genome that HAT automatically filters, we find high levels of overlap with DeepTrio, as well as confirmed unique DNV calls from HAT.

## 4 Discussion

DNV calling from multiple sequencing types is critical for studies of mutation rates and human disease. Several tools exist for calling DNVs from Illumina short-read sequencing data (Michaelson *et al.* 2012, Wei *et al.* 2014, Lian *et al.* 2021) including our recently developed tool HAT that works on short-read WGS data (Ng *et al.* 2022). However, we are only aware of one tool [DeepTrio (Kolesnikov *et al.* 2021)] that can call DNVs from Illumina short-read sequencing data and PacBio HiFi long-read sequencing data. In this study, we advanced our tool HAT to also work on these data types.

There are multiple advantages of using HAT for DNV calling including utility of workflow in the cloud, use of multiple underlying variant callers, and speed. HAT is designed to be implemented in several possible ways. In particular, the Cromwell implementation makes HAT cloud-friendly and compatible with the Terra platform (https://terra.bio/) on which several datasets from the National Institute of Health are hosted in the cloud (https://anvilproject.org). This should facilitate the use of HAT by multiple research groups. Unlike DeepTrio that works on data from one underlying variant caller, we make use of two underlying variant callers (i.e. GATK and DeepVariant). In our framework, the use of two underlying variant callers increases the specificity of the callset. Finally, our use of GPU acceleration facilitates accelerated DNV detection.

The well-known HG002 trio from the Genome In A Bottle project has been studied for many years and with several different sequencing technologies. We generated a HAT DNV callset for this trio from two sequencing technologies including Illumina short-read WGS and PacBio long-read WGS. This DNV resource will be helpful to others who are interested in trying out HAT in their own labs and for the greater research community interested in this trio for research purposes.

Currently, the majority of sequencing data utilized for assessing DNVs is from short-read sequencing platforms. However, we are at a juncture in genomics whereby highly accurate long-read sequencing data will become more commonplace and a method to assess DNVs in this type of data is critical. HAT works on Illumina short-read WES, Illumina short-read WGS, and PacBio HiFi long-read WGS. Future steps will integrate additional sequencing technologies as they improve accuracy (i.e. Oxford Nanopore Technology) and/or become more widely available (e.g. PacBio Onso, Element Biosciences, Singular Genomics).

Overall, HAT is a DNV caller that will be of interest to individuals studying DNVs for various purposes (e.g. mutation rates, human disease).

## Supplementary Material

btad775_Supplementary_Data

## Data Availability

The main HAT GitHub link is here: https://github.com/TNTurnerLab/HAT. The Simons Simplex Collection WGS data are available as described previously (Ng *et al.* 2022). The PB.100 and 9p.100 family datasets are available as described previously (Mehinovic *et al.* 2022, Sams *et al.* 2022). The Genome In A Bottle datasets are available at https://github.com/genome-in-a-bottle/giab_data_indexes.
