# Supplementary material for: HAT: de novo variant calling for highly accurate short-read and long-read sequencing data
Source: Bioinformatics. 2024 Jan 4;40(1):btad775. doi: 10.1093/bioinformatics/btad775 (PMC10777354; doi:10.1093/bioinformatics/btad775)
Supplement: btad775_Supplementary_Data [file btad775_supplementary_data.zip › HAT_supplemental_table_legends.docx]

**Supplemental Table 1 100 SPARK WES trios *de novo* variant callset (Please see tab “supplemental_table_1” of the supplemental_tables_12.4.2023.xlsx)**

Here are the HAT results from 100 trios taken from the SPARK collection. Each *de novo* variants (DNVs) is marked with a high and low confidence score and if it was confirmed or not by our filtering step.

**Supplemental Table 2 HG002 DNVs mastertable (Please see tab “supplemental_table_2” of the supplemental_tables_12.4.2023.xlsx)**

Here are the HAT and DeepTrio DNV results from multiple sequencing technologies of the HG002 trio. Included are the HAT Illumina WGS and PacBio HiFi long read data, as well as the DeepTrio results, run on Illumina WGS data, and HG002 truthset. The count data is derived from 300x Illumina WGS data.

**Supplemental Table 3 PacBio Long Read DNV callset (Please see tab “supplemental_table_3” of the supplemental_tables_12.4.2023.xlsx)**

Here are the HAT results from PacBio HiFi long read data from 4 different trios. One trio is from a previous publication on 9p- syndrome and 3 trios from a previous publications on autism.

**Supplemental Table 4 *de novo* variants detected in four families sequenced with long-read sequencing (Please see tab “supplemental_table_4” of the supplemental_tables_12.4.2023.xlsx)**

This table shows the number of detected DNVs for four long-read sequenced families. The blue indicates a greater amount of variants, red lower.

**Supplemental Table 5 Percent of confirmed *de novo* variants detected in four families sequenced with long-read sequencing (Please see tab “supplemental_table_5” of the supplemental_tables_12.4.2023.xlsx)**

This table shows the percent of confirmed DNVs for the four long-read sequenced families.  The blue indicates a high percentage, red lower. DNVs were manually verified via visual inspection of the reads.
